# Crystal structure of trirubidium citrate from laboratory X-ray powder diffraction data and DFT comparison

**DOI:** 10.1107/S2056989017001086

**Published:** 2017-01-27

**Authors:** Alagappa Rammohan, James A. Kaduk

**Affiliations:** a Atlantic International University, Honolulu HI, USA; b Illinois Institute of Technology, Chicago IL, USA

**Keywords:** crystal structure, powder diffraction, density functional theory, citrate, rubidium

## Abstract

The crystal structure of trirubidium citrate, 3Rb^+^·C_6_H_5_O_7_^3−^, has been solved and refined using laboratory X-ray powder diffraction data, and optimized using density functional techniques. The two independent Rb^+^ cations are seven- and eight-coordinate, with bond-valence sums of 0.99 and 0.92 valence units. The coordination polyhedra share edges and corners to form a three-dimensional framework. The only hydrogen bond is an intra­molecular one between the hy­droxy group and the central carboxyl­ate, with graph set *S*(5). The hydro­phobic methyl­ene groups lie in pockets in the framework.

## Abstract 2

The crystal structure of trirubidium citrate has been solved and refined using laboratory X-ray powder diffraction data, and optimized using density functional techniques.

## Chemical context   

In the course of a systematic study of the crystal structures of Group 1 (alkali metal) citrate salts to understand the anion’s conformational flexibility, ionization, coordination tendencies, and hydrogen bonding, we have determined several new crystal structures. Most of the new structures were solved using X-ray powder diffraction data (laboratory and/or synchrotron), but single crystals were used where available. The general trends and conclusions about the sixteen new compounds and twelve previously characterized structures are being reported separately (Rammohan & Kaduk, 2017*a* ▸). Eight of the new structures – NaKHC_6_H_5_O_7_, NaK_2_C_6_H_5_O_7_, Na_3_C_6_H_5_O_7_, NaH_2_C_6_H_5_O_7_, Na_2_HC_6_H_5_O_7_, K_3_C_6_H_5_O_7_, Rb_2_HC_6_H_5_O_7_, and Rb_3_C_6_H_5_O_7_(H_2_O) – have been published recently (Rammohan & Kaduk, 2016*a* ▸,*b* ▸,*c* ▸,*d* ▸,*e* ▸, 2017*b* ▸,*c* ▸; Rammohan *et al.*, 2016 ▸), and two additional structures – KH_2_C_6_H_5_O_7_ and KH_2_C_6_H_5_O_7_(H_2_O)_2_ – have been communicated to the CSD (Kaduk & Stern, 2016*a* ▸,*b* ▸).
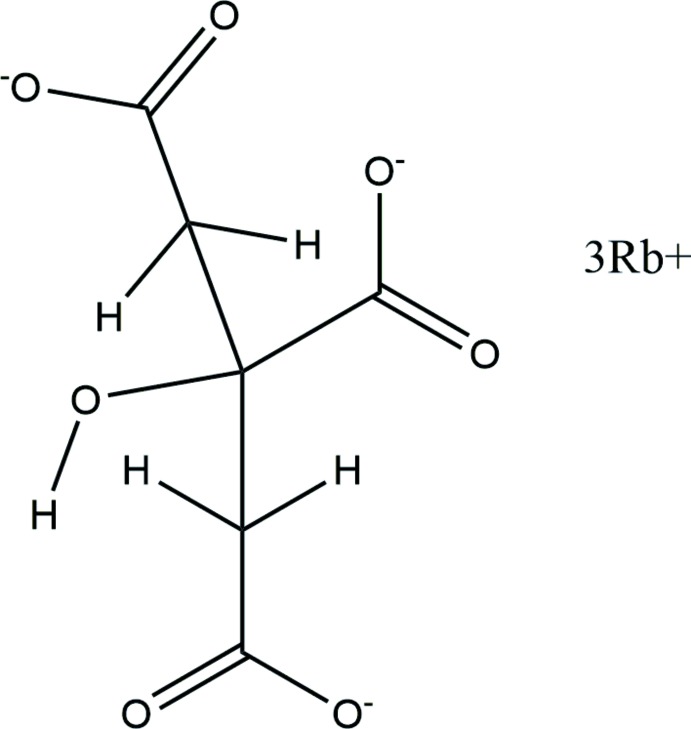


## Structural commentary   

**Figure 1:**
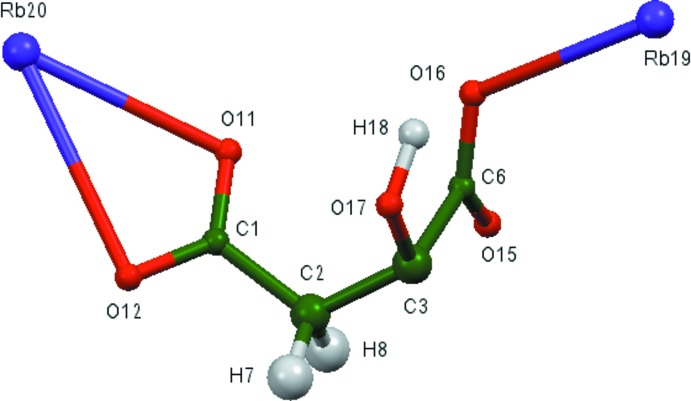
The asymmetric unit of trirubidium citrate, showing the atom numbering. The atoms are represented by 50% probability spheroids.

**Figure 2:**
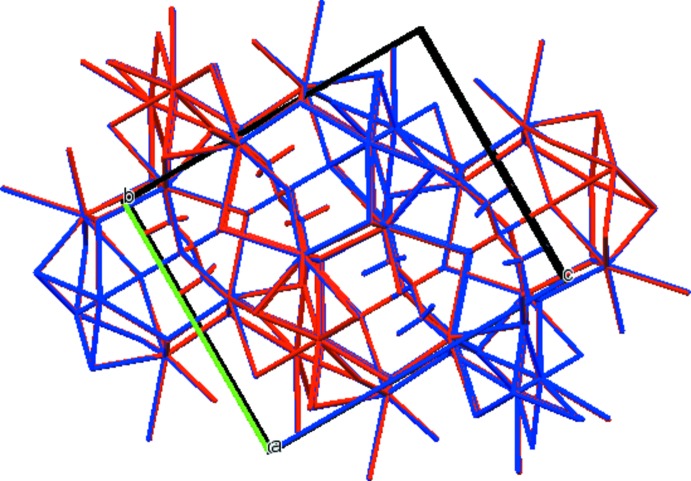
Comparison of the refined and optimized structures of trirubidium citrate. The refined structure is in red, and the DFT-optimized structure is in blue.

The asymmetric unit of the title compound is shown in Fig. 1 ▸. The root-mean-square deviation of the non-hydrogen atoms in the Rietveld-refined and DFT-optimized structures is 0.052 Å (Fig. 2 ▸). The largest difference is 0.086 Å, at C1. The excellent agreement between the two structures is strong evidence that the experimental structure is correct (van de Streek & Neumann, 2014 ▸). This discussion uses the DFT-optimized structure. Most of the bond lengths, bond angles, and torsion angles fall within the normal ranges indicated by a *Mercury* Mogul geometry check (Macrae *et al.*, 2008 ▸). The C3—C2—C1 angle of 116.5° is flagged as unusual [*Z*-score = 2.3; average = 112.7 (16)°]. The C2—C3—C2 angle of 106.0° is also flagged as unusual [*Z*-score = 2.5; average = 109.6 (14)°]. This hygroscopic compound was measured *in situ*, so perhaps slightly unusual geometry could be expected.

The citrate anion occurs in the *trans,trans*-conformation, which is one of the two low-energy conformations of an isolated citrate. The central carboxyl­ate group and the hy­droxy group lie on a mirror plane. The terminal carboxyl­ate O11 atom and the central carboxyl­ate O15 atom chelate to Rb19, O11 and the central carboxyl­ate O16 atom chelate to a second Rb19, and the terminal carboxyl­ate O12 atom and the O17 hy­droxy group chelate to a third Rb19. The terminal O11–C1–C12 carboxyl­ate group acts as a bidentate ligand to Rb20. The Mulliken overlap populations and atomic charges indicate that the metal-oxygen bonding is ionic.

The Bravais–Friedel–Donnay–Harker (Bravais, 1866 ▸; Friedel, 1907 ▸; Donnay & Harker, 1937 ▸) morphology suggests that we might expect blocky morphology for trirubidium citrate, with {011} as the principal faces. A 4th-order spherical harmonic texture model was included in the refinement. The texture index was 1.001, indicating that preferred orientation was not significant for this rotated flat-plate specimen.

## Supra­molecular features   

**Figure 3:**
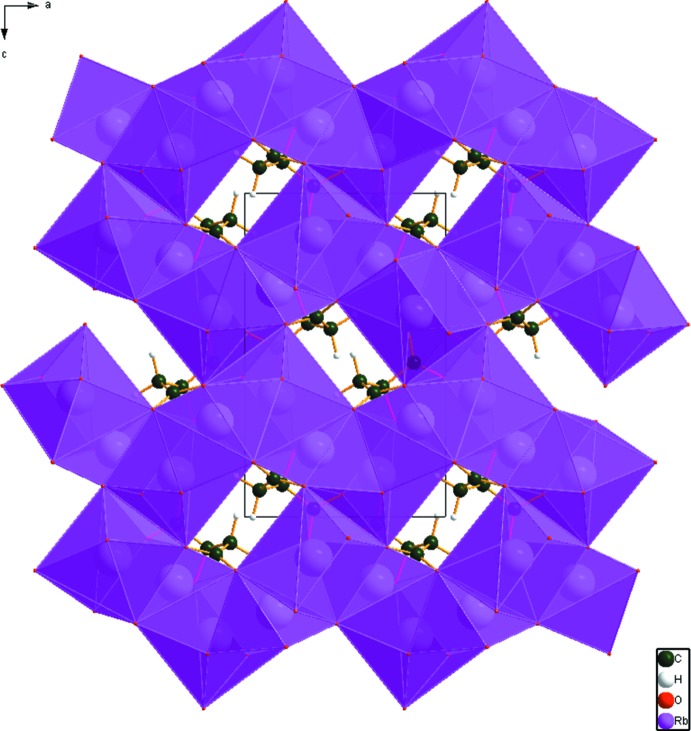
Crystal structure of trirubidium citrate, viewed down the *b* axis.

The two independent Rb^+^ cations, Rb19 and Rb20, are seven- and eight-coordinate, with bond-valence sums of 0.99 and 0.92 valence units, respectively. The coordination polyhedra share edges and corners to form a three-dimensional network (Fig. 3 ▸). The only hydrogen bond is an intra­molecular one (Table 1 ▸) between the hy­droxy group and the central carboxyl­ate, with graph set *S*(5). The Mulliken overlap population indicates, by the correlation in Rammohan & Kaduk (2017*a* ▸), that this hydrogen bond contributes 12.6 kcal mol^−1^ to the crystal energy.

## Database survey   

Details of the comprehensive literature search for citrate structures are presented in Rammohan & Kaduk (2017*a* ▸). A reduced cell search of the cell of trirubidium citrate monohydrate in the Cambridge Structural Database (Groom *et al.*, 2016 ▸) (increasing the default tolerance from 1.5 to 2.0%) yielded 221 hits, but combining the cell search with the elements C, H, O, and Rb only yielded no hits.

## Synthesis and crystallization   

A portion of Rb_3_(C_6_H_5_O_7_)(H_2_O)_1_ (Rammohan & Kaduk, 2017*c* ▸) was heated at 14 K min^−1^ to 463 K and held at that temperature for 10 min. The white solid was immediately transferred to a glass vial to cool.

## Refinement   

**Figure 4:**
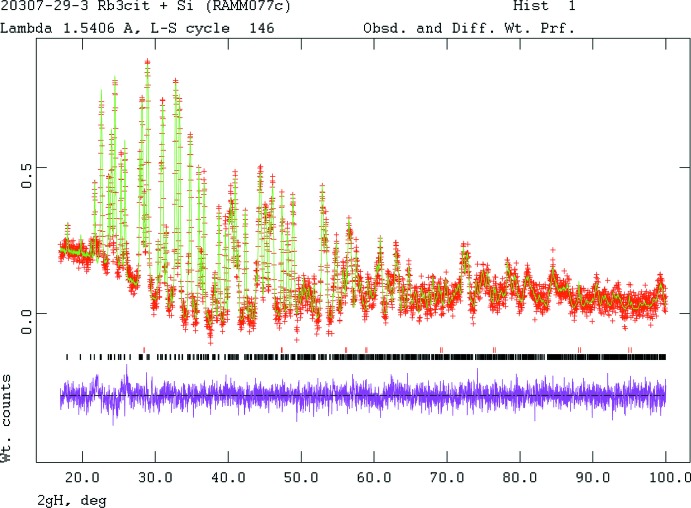
Rietveld plot for the refinement of trirubidium citrate. The vertical scale is not the raw counts but the counts multiplied by the least-squares weights. This plot emphasizes the fit of the weaker peaks. The red crosses represent the observed data points, and the green line is the calculated pattern. The magenta curve is the difference pattern, plotted at the same scale as the other patterns. The row of black tick marks indicates the reflection positions. The red tick marks indicate the positions of the peaks of the Si inter­nal standard.

Crystal data, data collection and structure refinement details are summarized in Table 2 ▸. Diffraction data are displayed in Fig. 4 ▸. The white solid was ground in a mortar and pestle, blended with NIST 640b Si inter­nal standard in order to verify the calibrated goniometer zero error, packed into a standard Bruker D2 sample cell and protected from the atmosphere by an 8 µm thick Kapton window attached to the cell with Vaseline. The powder pattern indicated that the sample was still hydrated, so the blend was re-heated at 17 K min^−1^ to 483 (10) K and held for 10 min. Re-measuring the powder pattern indicated that a new phase had formed.

The pattern was indexed using *DICVOL06* (Louër & Boultif, 2007 ▸) on a primitive ortho­rhom­bic cell having *a* = 7.904, *b* = 12.701, *c* = 10.773 Å, and *V* = 1081.8 Å^3^. These lattice parameters are 2.6, 1.8, and 3.3% larger than those of K_3_C_6_H_5_O_7_ (Rammohan & Kaduk, 2016*e* ▸), and the volume is 7.9% larger. The compound was assumed to be isostructural to the K analogue (space group *Pna2_1_*), and the coordinates of tripotassium citrate were used as the initial model for the Rietveld refinement.

Pseudo-Voigt profile coefficients were as parameterized in Thompson *et al.* (1987 ▸) with profile coefficients for Simpson’s rule integration of the pseudo-Voigt function according to Howard (1982 ▸). The asymmetry correction of Finger *et al.* (1994 ▸) was applied, and microstrain broadening by Stephens (1999 ▸). The structure was refined by the Rietveld method using *GSAS/EXPGUI* (Larson & Von Dreele, 2004 ▸; Toby, 2001 ▸). All C—C and C—O bond lengths were restrained, as were all bond angles. The hydrogen atoms were included at fixed positions, which were recalculated during the course of the refinement using *Materials Studio* (Dassault Systèmes, 2014 ▸). The *U*_iso_ value of the C atom in the central part of the citrate anion, and the C and O atoms on the exterior, were constrained to be equal, and the *U*_iso_ valuess of the hydrogen atoms were constrained to be 1.3 times those of the atoms to which they are attached.

The structure refined satisfactorily (*R*_wp_ = 0.0301 and reduced χ^2^ = 1.828 for 69 variables) in space group *Pna*2_1_ (the space group of the K analogue), but both the ADDSYM module of *PLATON* (Spek, 2009 ▸) and the Find Symmetry module of *Materials Studio* (Dassault Systèmes, 2014 ▸) suggested the presence of an additional centre of symmetry, and that the space group was *Pnma* (with a transformation of axes). The tolerance on the search was 0.12 Å. Because lower residuals were obtained with fewer parameters, we believe that *Pnma* is the correct space group.

## DFT calculations   

After the Rietveld refinement, a density functional geometry optimization (fixed experimental unit cell) was carried out using *CRYSTAL14* (Dovesi *et al.*, 2014 ▸). The basis sets for the C, H, and O atoms were those of Peintinger *et al.* (2012 ▸), and the basis set for Rb was that of Schoenes *et al.* (2008 ▸). The calculation was run on eight 2.1 GHz Xeon cores (each with 6 Gb RAM) of a 304-core Dell Linux cluster at IIT, used 8 *k*-points and the B3LYP functional, and took about seven h. The *U*_iso_ values from the Rietveld refinement were assigned to the optimized fractional coordinates.

## Supplementary Material

Crystal structure: contains datablock(s) RAMM077C_publ, ramm077c_DFT, RAMM077C_overall, RAMM077C_phase_1, RAMM077C_phase_2, RAMM077C_p_01. DOI: 10.1107/S2056989017001086/vn2123sup1.cif

Supporting information file. DOI: 10.1107/S2056989017001086/vn2123RAMM077C_phase_1sup2.cml

Supporting information file. DOI: 10.1107/S2056989017001086/vn2123RAMM077C_phase_2sup3.cml

CCDC references: 1529083, 1529084, 1529085

Additional supporting information:  crystallographic information; 3D view; checkCIF report

## Figures and Tables

**Table 1:** Hydrogen-bond geometry (Å, °) for ramm077c_DFT[Chem scheme1]

*D*—H⋯*A*	*D*—H	H⋯*A*	*D*⋯*A*	*D*—H⋯*A*
O17—H18⋯O16	0.981	1.862	2.572	126.7

**Table 2:** Experimental details

Crystal data	
Chemical formula	3Rb^+^·C_6_H_5_O_7_^3−^
*M*_r_	445.50
Crystal system, space group	Orthorhombic, *P* *n* *m* *a*
Temperature (K)	300
*a*, *b*, *c* (Å)	7.9096 (2), 10.7733 (3), 12.6986 (3)
*V* (Å^3^)	1082.08 (7)
*Z*	4
Radiation type	*K*α_1_, *K*α_2_, λ = 1.540593, 1.544451 Å
Specimen shape, size (mm)	Flat sheet, 24 × 24

Data collection	
Diffractometer	Bruker D2 Phaser
Specimen mounting	Standard PMMA holder with Kapton window
Data collection mode	Reflection
Scan method	Step
2θ values (°)	2θ_min_ = 5.00 2θ_max_ = 100.01 2θ_step_ = 0.020

Refinement	
*R* factors and goodness of fit	*R*_p_ = 0.020, *R*_wp_ = 0.025, *R*_exp_ = 0.023, *R*(*F* ^2^) = 0.048, χ^2^ = 1.232
No. of parameters	57
No. of restraints	13

The same symmetry and lattice parameters were used for the DFT calculation. Computer programs: *DIFFRAC.Measurement* (Bruker, 2009 ▸), *GSAS* (Larson & Von Dreele, 2004 ▸), *DIAMOND* (Crystal Impact, 2015 ▸) and *publCIF* (Westrip, 2010 ▸).

## supplementary crystallographic information

### Crystal data

**Table:**

C_6_H_5_O_7_Rb_3_	*b* = 10.7733 Å
*M**_r_* = 445.50	*c* = 12.6986 Å
Orthorhombic, *P**n**m**a*	*V* = 1082.08 Å^3^
*a* = 7.9096 Å	*Z* = 4

### Data collection

**Table:**

*h* = →	*l* = →
*k* = →	

### Fractional atomic coordinates and isotropic or equivalent isotropic displacement parameters (Å^2^)

**Table:**

	*x*	*y*	*z*	*U*_iso_*/*U*_eq_	
C1	0.85652	0.51237	0.11762	0.01290*	
C2	0.92710	0.63666	0.08156	0.02780*	
H7	1.04404	0.65330	0.12425	0.03610*	
H8	0.95879	0.63156	−0.00199	0.03610*	
O11	0.70573	0.48606	0.09469	0.01290*	
O12	0.95620	0.44175	0.16822	0.01290*	
Rb19	0.34329	0.97183	0.12807	0.02550*	
C3	0.81408	0.75000	0.09868	0.02780*	
C6	0.65846	0.75000	0.02369	0.01290*	
O15	0.68502	0.75000	−0.07431	0.01290*	
O16	0.51559	0.75000	0.06782	0.01290*	
O17	0.75259	0.75000	0.20645	0.01290*	
H18	0.62975	0.75000	0.19609	0.01680*	
Rb20	0.14960	0.25000	0.29368	0.02550*	

### Bond lengths (Å)

**Table:**

C1—C2	1.521	C3—C2^i^	1.529
C1—O11	1.260	C3—C6	1.556
C1—O12	1.270	C3—O17	1.452
C2—C3	1.529	C6—O15	1.262
C2—H7	1.087	C6—O16	1.261
C2—H8	1.092	O17—H18	0.981

Symmetry code: (i) *x*, −*y*+3/2, *z*.

### Hydrogen-bond geometry (Å, º)

**Table:**

*D*—H···*A*	*D*—H	H···*A*	*D*···*A*	*D*—H···*A*
O17—H18···O16	0.981	1.862	2.572	126.7
